# Using biomarkers prospectively in adaptive clinical trials

**DOI:** 10.1186/1745-6215-12-S1-A14

**Published:** 2011-12-13

**Authors:** Donald A Berry

**Affiliations:** 1 M.D. Anderson Cancer Center, Houston, TX, USA

## 

A standard approach to biomarker research is to retrospectively examine interactions between biomarkers and treatment effects. An alternative approach of examining such interactions prospectively has advantages. First, if the biomarker information is required for randomization then eliminates missing biomarker information. Another is that nonresponding biomarker subtypes in a multiarmed trial that do not respond to a particular arm can be excluded from that arm, perhaps gradually, using adaptive randomization. A consequence of adaptively excluding nonresponders is the potential to have a smaller, more focused trial.

There are limitations to such an approach. One is that the biomarker has to be available to enable adapting to the accumulating evidence. Another is that the outcome database must be updated in a reasonably timing fashion, and it must be connected it to the patient assignment algorithm. In addition, the design, although fully prospective, is complicated to convey to investigators, patients, and IRBs, IECs, and other regulators.

A third possible design is to restrict trial eligibility to the population that is the drug’s target. This is efficient, but it relies on knowing the target. This design gives no information about biomarker by treatment interactions. And it inhibits learning about the roles of other biomarkers.

The adaptive approach is a compromise between the first and third approaches: Start with all-comers but restrict to the responding population as the trial results accumulate.

The approaches I describe employ randomization. All have an adaptive aspect in which accumulating trial results are analyzed frequently with the possibility of modifying the trial’s future course. Many treatment arms are possible, including combination therapies. So it is possible to learn about the way treatments interact with each other as well as the way they interact with biomarkers.

Having multiple biomarkers and multiple treatment arms increases the false-positive rate. Therefore it is essential to prospectively build some level of confirmation into the design.

False-positive rates and statistical power can be evaluated by simulation and controlled.

Taking an adaptive approach is fruitless without information to which to adapt. There is little information available when the endpoints are long-term. However, early markers of therapeutic effect (longitudinal biomarkers, measurements of tumor burden, etc.) can be correlated with long-term clinical outcome.

I will give an example (called I-SPY 2) of an adaptive biomarker-driven trial in neoadjuvant breast cancer. The goal is to efficiently identify biomarker signatures for a variety of agents and combinations being considered simultaneously.

